# Effects of ibrutinib on T-cell immunity in patients with chronic lymphocytic leukemia

**DOI:** 10.3389/fimmu.2022.962552

**Published:** 2022-08-19

**Authors:** Yanyan Liu, Yongping Song, Qingsong Yin

**Affiliations:** Department of Hematology, The Affiliated Cancer Hospital of Zhengzhou University and Henan Cancer Hospital, Zhengzhou, China

**Keywords:** chronic lymphocytic leukemia, ibrutinib, T-cell immunity, tumor microenvironment, immune regulation

## Abstract

Chronic lymphocytic leukemia (CLL), a highly heterogeneous B-cell malignancy, is characterized by tumor microenvironment disorder and T-cell immune dysfunction, which play a major role in the proliferation and survival of CLL cells. Ibrutinib is the first irreversible inhibitor of Bruton’s tyrosine kinase (BTK). In addition to targeting B-cell receptor (BCR) signaling to kill tumor cells, increasing evidence has suggested that ibrutinib regulates the tumor microenvironment and T-cell immunity in a direct and indirect manner. For example, ibrutinib not only reverses the tumor microenvironment by blocking cytokine networks and toll-like receptor signaling but also regulates T cells in number, subset distribution, T-cell receptor (TCR) repertoire and immune function by inhibiting interleukin-2 inducible T-cell kinase (ITK) and reducing the expression of inhibitory receptors, and so on. In this review, we summarize the current evidence for the effects of ibrutinib on the tumor microenvironment and cellular immunity of patients with CLL, particularly for the behavior and function of T cells, explore its potential mechanisms, and provide a basis for the clinical benefits of long-term ibrutinib treatment and combined therapy based on T-cell-based immunotherapies.

## Introduction

Chronic lymphocytic leukemia (CLL) is a malignancy of small, mature B lymphocytes that clonally expand into secondary lymphoid organs, bone marrow, and peripheral blood, resulting in lymphadenopathy, splenomegaly, and hematopoietic failure (1, 2). Tumor microenvironment disorder and T-cell immune dysfunction are prominent characteristics of CLL that are clinically manifested as increased susceptibility to opportunistic infections such as viruses and fungi and an increased incidence of autoimmune diseases and secondary malignant tumors (2), which are also the main causes of failure of T-cell-based immunotherapies and drug resistance (3, 4). T cells in CLL patients, as major supporting cells in the tumor microenvironment and particularly CD4^+^ T cells, nourish CLL cells through complex cytokine networks or direct contact (5). Moreover, CD8^+^ T cells demonstrate an “exhausted” phenotype with progressive loss of effector function and impaired memory T-cell potential (6, 7). Therefore, reversing microenvironment disorders and reconstituting T-cell immunity may be critical to improving the outcome of CLL patients (8, 9).

The B-cell receptor (BCR) signaling pathway in CLL cells is reportedly overactivated; thus, targeting the key kinases of the BCR pathway is a promising anti-leukemia therapy. BCR signaling is initiated through upstream kinases, including SYK, BTK, and PI3K, and these can be inhibited by corresponding small-molecule kinase inhibitors (10). First-generation BTK inhibitors (BTKis), such as ibrutinib, acalabrutinib, and zanubrutinib, are irreversible at the C418 site of BTK (11). Second-generation BTKis, such as fenibrutinib, vecabrutinib, and nembrolizumab, can reversibly inhibit BTK and to some extent overcome the drug resistance of first-generation BTKis (11). Ibrutinib, as the first BTKi, has profoundly altered the treatment paradigm of CLL patients, particularly relapsed/refractory CLL (R/R CLL) and high-risk patients with TP53 aberrations (12–14). Both acalabrutinib and zanubrutinib have demonstrated higher selectivity and fewer off-target effects than ibrutinib (11, 15, 16). Nevertheless, previous studies have shown that ibrutinib not only inhibits BCR and nuclear factor kappa B (NF-κB) signaling (17–19) but also plays multiple roles in regulating the tumor microenvironment and T-cell immunity in CLL patients (20–22). This activity has been confirmed by significant improvement in the efficacy of CAR-T cells and the bispecific antibody blinatumomab in the clinic (3, 23–25). However, to date, the effect of ibrutinib on the microenvironment and T-cell immunity of patients with CLL is not completely clear.

Therefore, this article reviews the effects of ibrutinib on the microenvironment and cellular immunity of patients with CLL, particularly on the behavior and function of T cells, and their potential mechanisms, to provide a basis for the clinical benefits of long-term ibrutinib treatment and the further design of combined therapy based on T-cell-based immunotherapies.

## Overall regulation of ibrutinib on CLL microenvironment

**Figure 1:**
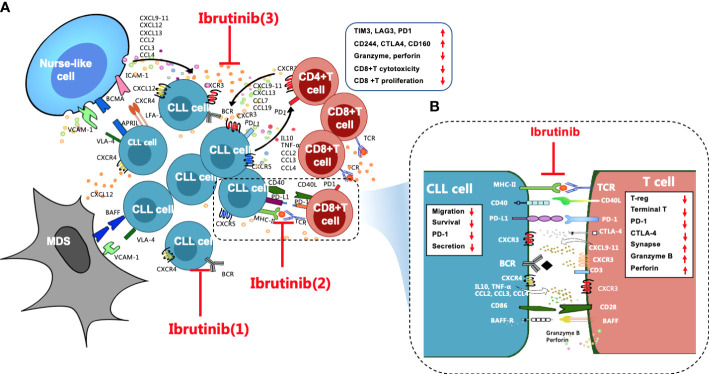
Overall regulation of ibrutinib on the CLL microenvironment, particularly T cell immunity, is important. **(A)** Stromal cells, NLCs, and T cells are the three key supporting cells in the CLL microenvironment. They crosstalk with CLL cells through direct contact and chemokines/cytokines and their ligand-receptor interactions, such as CXCR4/CXCL12, CXCR3/CXCL9,10,11, and CXCR5/CXCL13, to mediate the activation, homing, proliferation, and survival of CLL cells, which leads to T-cell immune dysfunction, particularly for CD8^+^ T cells, which are excessively activated, expanded, and gradually pseudo-exhausted. Exhausted CD8^+^ T cells highly express a variety of inhibitory receptors, such as PD1, CTLA4, CD244, TIM3, and LAG3. The cytotoxicity and proliferation activity of CD8^+^ T cells decrease. Ibrutinib regulates the CLL microenvironment by (1) blocking BCR signaling, (2) preventing direct contact between CLL cells and T cells and repairing impaired immune synapses, and (3) inhibiting cytokine networks. **(B)** These effects contribute to improving the activity of effector T cells in CLL patients, such as increased granzyme B and perforin secretion, reduced inhibitory receptors, etc. In addition, ibrutinib inhibits the cytokine secretion, migration, proliferation, and survival of CLL cells.

Secondary lymphoid organs, which are also called proliferation centers, have a more complex microenvironment that is more conducive to CLL cell survival (18), and this is where the BCR signaling activity of CLL cells is upregulated, and the proliferative activity of CLL cells is also increased (18, 26). Stromal cells, nurse-like cells (NLCs), and T cells are three supporting cells in the CLL microenvironment (27) (**Figure 1A**) that mediate the activation, homing, proliferation, and survival of CLL cells *via* direct contact and the secretion of chemokine/cytokines and adhesion molecules as well as their ligand–receptor interactions (28–33). Together, these constitute complex cytokine networks (33, 34) that in turn recruit the migration of T cells (35), including Th2 cells and Tregs, and induce T–cell immune tolerance, T-cell anergy, and the immune escape of CLL cells (36, 37). Additionally, toll-like receptors (TLRs) interact with BTK, connecting the BCR signal with TLR signals and activating the NF-кB signaling pathway, eventually promoting the proliferation and survival of CLL cells (38, 39). Moreover, TLR signaling also increases the immune escape of CLL cells by inducing Treg expansion and producing immunosuppressive molecules (40).

Accumulating studies have demonstrated that ibrutinib regulates the disordered microenvironment in CLL patients (18, 34). Specifically, ibrutinib directly inhibits the activation and proliferation of CLL cells by blocking the BCR and the NF-κB signaling pathways (18, 19, 31). Moreover, ibrutinib blocks the close “crosstalk” between CLL cells and supporting cells in the microenvironment to prevent their protection of CLL cells by blocking complex cytokine networks and direct contact (27, 39). For instance, ibrutinib not only inhibits the secretion of cytokines, such as CCL3, CCL4, CXCL12, and CXCL13, from CLL cells and their supporting cells within the microenvironment, it also inhibits the TLR signaling pathway (38, 41), which prevents the homing and residence of CLL cells and dissociates these cells from the protective microenvironment (31, 41, 42).

## The effects of ibrutinib on circulating T-cell counts

**Figure 2:**
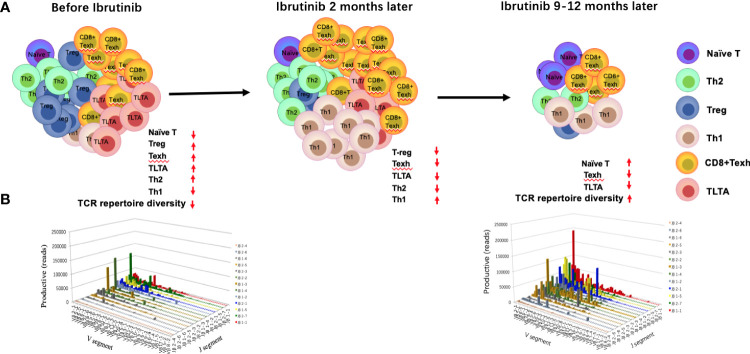
Changes in the T-cell compartment and T-cell repertoire before and during ibrutinib treatment. The absolute numbers and percentage of CD4^+^ and CD8^+^ T cells increase in the peripheral blood, particularly for CD8^+^ T cells. However, the distribution of T-cell subgroups is abnormal, which leads to the imbalance of Th1/Th2, an increase in T-regs, long-term activated T cells (TLTAs), and terminally differentiated T cells such as effector memory T cells (T_EM_) and exhausted T cells (Texh cells), and a decrease in naïve T cells (A, left penal). There is a severely skewed T-cell repertoire in patients with CLL **(B)**. After 1–2 months of ibrutinib treatment, the number of T cells demonstrates a transient increase and then decreases gradually after 6 months (**(A)** middle penal). The distribution of the T-cell subgroups is near to the normal level at 12 months, in parallel with partial reconstruction of the T-cell repertoire diversity [**(A)** right penal; **(B)**].

Increasing studies have shown that CD4^+^ and CD8^+^ T cells are enriched in the peripheral blood of CLL patients (43). Specifically, the absolute numbers of CD3^+^, CD4^+^, and CD8^+^ T cells significantly increased in the peripheral blood of R/R and naïve CLL patients before ibrutinib treatment (22, 28, 34, 44, 45), particularly CD8^+^ T cells (20, 44), which resulted in a decreased CD4:CD8 ratio (28, 45) (**Figure 2A**).

Burger and colleagues described for the first time that ibrutinib treatment induces lymphocyte redistribution using 2H-labeling experiments, which results in increased absolute lymphocyte counts in peripheral blood and significant shrinkage of lymph nodes (46). Subsequently, numerous studies have focused on the effects of ibrutinib on the number of T cells and distribution of T-cell subpopulations, but these results remain controversial. Parry et al. found that CD3^+^ and CD8^+^ T-cell counts significantly decreased in R/R and naïve CLL patients who were treated with ibrutinib for 6 months (47). Niemann et al. found that the proportion of CD4^+^ and CD8^+^ T cells decreased dramatically after 6 months (34). Yin et al. found that the increased CD3^+^, CD4^+^, and CD8^+^ T cells were significantly decreased in R/R CLL patients after 3 months of ibrutinib treatment and then dropped to the normal range after 12 months (22). However, Long et al. noted a progressive increase in T-cell counts until 6 months of ibrutinib treatment in R/R and primary CLL patients (21). Single-cell sequencing also showed that the percentage of T-cell subgroups changed after treatment with ibrutinib, particularly the percentage of CD8^+^T cells, which increased gradually until 4 months, while CD4^+^T cells decreased gradually, which coincided with the progressive reduction of CLL cells (20). Differences in the above results may be related to disease status, tumor burden, and time point after ibrutinib treatment. Recently, a study with more intensive detection time points demonstrated there was a transient increase in the T-cell number in CLL patients at about 2 months after ibrutinib treatment, followed by a decrease after 6–10 months that gradually returned to normal levels at 1 year (42).

The reason for the transient increase in T-cell counts after ibrutinib treatment is completely unclear. It may be related to the redistribution of T and CLL cells to the peripheral blood after ibrutinib treatment (48, 49), followed by a simultaneous decline in both T and CLL cells (22, 47–50). Additionally, ibrutinib can reduce activation-induced cell death (AICD) by inhibiting interleukin-2 inducible T cell kinase (ITK) activity, causing a short-term increase in circulating T-cell counts (21). Sun et al. also confirmed this finding in an ITK-deficient mouse model (51).

## The regulation of ibrutinib on the differentiation of T-cell subpopulations

**Figure 3:**
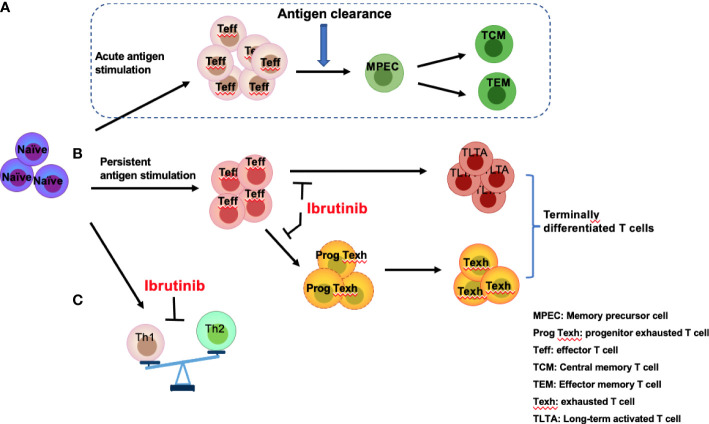
Effects of ibrutinib on effector T-cell subgroups. Antigen stimulation drives naïve T cells to differentiate into effector T cells (Teff) followed by rapid expansion of effector T cells, which eventually produce memory precursor cells (MPECs) after antigen clearance, further differentiating into central memory T cells (TCM) and effector memory T cells (TEM) **(A)**. However, persistent antigen stimulation from infections or CLL-related antigens induces over-activation of T cells and promotes progenitor exhausted T (Prog Texh) cells to differentiate into exhausted T (Texh) cells, which leads to the accumulation of terminally differentiated T cells, including TLTA and Texh cells **(B)**. In addition, Th1 and Th2 differentiation is unbalanced **(C)**. Ibrutinib reverses the pseudo-exhaustion of T cells and promotes the activity of effector T cells and the predominant differentiation of Th1 cells in CLL patients.

T cells are key adaptive immune effector cells that can be mainly classified into multi-functional helper T cells (Th cells), immunosuppressive Tregs, and cytotoxic T cells (CTLs). Acute antigen stimulation drives naïve T-cell differentiation and rapid expansion of effector T cells (**Figure 3A**). However, prolonged exposure to an antigen from chronic viral infections or cancer induces exhaustion in the responding CD8^+^ effector T-cell populations (52). Moreover, the differentiation of T-cell subgroups is unbalanced in patients with CLL (**Figures 3B** **,** **C**).

### Th1 and Th2 cells

In CLL patients, TCR signaling activation triggers a signaling cascade to activate ITK, promoting the differentiation of Th2 and Treg cells and inhibiting the differentiation of Th1 and cytotoxic CD8^+^ T cells (53, 54). Additionally, a series of cytokines/chemokines, such as IL6, IL4, and IL10, which are produced by CLL and Th2 cells, can promote immune system skewing toward Th2 cells and inhibit Th1 differentiation (55) (**Figure 3C**). Therefore, compared with healthy subjects, the number of Th1 and Th2 cells in CLL patients increases (56), but the distribution is unbalanced, demonstrating the dominant differentiation of Th2 cells (53), which results in a tumor-promoting environment and a reduced role for Th1 in tumor monitoring (54, 57). Several studies have shown that ITK deficiency promotes T-bet expression and blocks ITK-dependent Th2 cell differentiation (53, 58, 59), while Th1 cell differentiation can be triggered by the substitution of another lymphocyte kinase (RLK) (59). Therefore, ibrutinib can irreversibly bind to ITK to suppress Th2 cell differentiation, restoring the balance between Th1 and Th2 (53).

### Tregs and Th17 cells

Tregs and IL-17-producing CD4^+^ (Th17) cells play an important role in immune tolerance and homeostasis (8, 60). In CLL, Tregs may facilitate a tumor-promoting microenvironment and tumor progression (61, 62). Nevertheless, Th17 cells in anti-tumor immunity are negatively correlated with Tregs and positively correlated with invariant natural killer T cells (NKTs) (63). Disease progression in CLL patients and in the Eμ-TCL1 CLL mouse model is usually accompanied by a reduction in Th17 cells and Treg expansion and its immunosuppressive effector function (64–66), which demonstrates the different roles of these T cell subsets in CLL proliferation and survival. It has been reported that the frequency and absolute number of Tregs are significantly higher in CLL patients, which is related to high tumor burden and advanced disease (66, 67), and an increase in Tregs also indicates shorter overall survival (OS) (66). In contrast, Tregs were more suppressive in CLL patients than in healthy individuals, and the decrease in Tregs contributed to effective anti-tumor effects in animal models (68, 69). Fortunately, the absolute count and percentage of Treg were significantly reduced in CLL patients as early as 30 days after ibrutinib treatment (70). Animal models also revealed the effects of ibrutinib on Treg differentiation (71).

Th17 cells are an important component in CLL anti-tumor immune monitoring (72). Most studies have reported that a high Th17 cell number is positively associated with OS and negatively related to disease progression (65, 73–76), suggesting that Th17 has anti-tumor effects in CLL. Jadidi-Niaragh and colleagues found that CLL patients, including progressive and indolent patients, had a significantly lower frequency of Th17 cells compared with healthy subjects (65), which was consistent with the finding from Yousefi et al. (61). However, other studies also found that the frequency and absolute count of Th17 cells increased in CLL patients (56, 73, 74), particularly at early disease stages (8, 63). A previous study has demonstrated that the Th17 cells increased in patients with ibrutinib treatment by decreasing the FAS-mediated AICD (21). Moreover, ibrutinib reduced the number of Tregs, which also contributed to the expansion of Th17 cells to maintain the balance between Th17 cells and Tregs (73). However, contradictory findings, with decreased frequency and absolute number of Th17 T cells in patients with CLL receiving ibrutinib, have been reported (34, 55), and the possible reason was that ibrutinib impaired Th17 differentiation (77). It is speculated that these contradictory findings may be associated with the difference in the timing of detection and previous treatment history (3).

### Naïve T cells and terminally differentiated T cells

Patients with CLL experience abnormal T-cell differentiation due to persistent antigen stimulation from tumor antigens or infections, leading to over-activation of T cells with high expression of the activation markers CD38 and HLA-DR and the accumulation of long-term activated T cells (TLTAs) and terminally differentiated T cells (78, 79). There is also an increase in effector memory T cells (T_EM_), CD45RA-positive memory effector T cells (T_EMRA_), and exhausted T cells (Texh cells) (**Figures 2A**, **3C**), particularly during disease progression (45). Additionally, a significant decrease in naïve T cells and central memory T cells (T_CM_) (80), largely limits the immune function of T cells (45). Moreover, TLTAs reportedly maintain the ability to secrete cytokines and remain in a state of pseudo-exhaustion (50). These TLTAs and Texh cells are characterized by progressive loss in effector function, poor proliferative capacity (81) and upregulation of multiple inhibitory receptors, such as CD160, CD244, PD-1, TIM3, and cytotoxic T lymphocyte antigen 4 (CTLA-4) (44, 50, 81–83).

Accumulating data have demonstrated that ibrutinib can directly inhibit the expression of inhibitory receptors (21, 44, 50, 82, 84) and reduce the number of terminally differentiated T cells, such as TLTA, Texh, T_EM_, and T_EMRA_ cells (79, 84) but remain naïve T cells (79). Niemann et al. found that PD-1 expression significantly decreased at 4 weeks after ibrutinib treatment in CLL patients (34). Similarly, Solman et al. found that increased TLTA and Texh cell pre-treatment gradually decreased in R/R and primary CLL patients; specifically, the number of TLTA cells was significantly reduced after 2 months of ibrutinib and near normal at 9 months, and Texh cells decreased to the normal range at 5 months after ibrutinib treatment (79). Furthermore, studies have found that naïve CD4^+^ T cells increase in CLL patients with ibrutinib treatment (21, 79), and a large proportion of naïve T cells in CLL (greater than 10%) express T-BET or EOMES (21), indicating the differentiation is skewed to Th1 cells. Taken together, ibrutinib can inhibit the pseudo-exhaustion of T cells, thus reducing the number of TLTA and Texh cells and reversing the unbalanced T cell subgroups.

### Unconventional T cells

Unconventional T cells mainly include γδ T cells, NKT cells, and mucosal associated invariant T (MAIT) cells. γδ T cells account for about 10% of the T cells in peripheral blood, and 90% of γδ T cells carry the Vγ9Vδ2 TCR, which recognizes natural killer group 2D (NKG2D) and modulates γδ T-cell-driven immune responses (85). In CLL patients, both the absolute count and percentage of γδ T cells significantly increased (86), particularly the phenotype and function of Vγ9Vδ2-T cells changed. Specifically, compared with healthy individuals, CLL-derived Vγ9Vδ2 T cells were in a higher state of differentiation and had a lower ability to produce cytokines and degranulate, resulting in impaired granzyme-dependent cytotoxicity (87, 88). Ibrutinib treatment restored their function and cytotoxicity (88), which may be associated with the fact that ibrutinib promotes the phenotype of Vδ2Vγ9 T cells skewing toward Th1 cells in CLL patients by inhibiting ITK (87, 88).

In addition, NKT cells play a key role in regulating anti-tumor immunity (89). The frequency of NKT cells decreased with the development of CLL and could be a marker for immune monitoring and prognosis in CLL (90, 91). However, other studies found an increased absolute count of NKT cells in untreated CLL patients (42, 79), and a transient continuous increase at 3 months of ibrutinib treatment (79), and then the number of NKT cells displayed a gradual decrease and reached normal levels after 1 year (79).

MAIT cells exist in the liver and mucosal tissues, accounting for 1%–10% of peripheral blood T cells. MAIT cells are mainly involved in antibacterial immunity by producing a series of cytokines and lytic molecules, but little is known about their role in tumor immunity (92). Wallace et al. reported MAIT cell deficiency in CLL (93). However, so far, the regulatory effect of ibrutinib on MAIT cells remains unclear.

## The effects of ibrutinib on T cell functions

Generally, T cells are the key effector cells in anti-leukemia immunity. Nevertheless, in CLL patients, CD4^+^ T cells stimulate CLL cell survival and proliferation by secreting multiple chemokines/cytokines and through direct contact with the CD40 ligand (27, 36, 94), and CD8^+^ T cells are persistently activated and expanded within the CLL microenvironment and gradually become pseudo-exhausted (5, 50, 52), finally resulting in T-cell immune tolerance and the loss of their anti-tumor activity (27), which has been reported to be causative of the poor response to CAR-T cell therapies for CLL patients (95–97). Additionally, due to the damaged structure of effector T cells and the poor antigen presentation function of CLL cells, the formation of immune synapses between T and CLL cells is impaired (84, 98).

Clinical studies have found that ibrutinib regulates T-cell immunity through various mechanisms (**Figure 1B**). For instance, ibrutinib promotes immune synapse formation between T and tumor cells and restores immune function by enhancing F-actin polarization and protein tyrosine phosphorylation (99, 100). Moreover, long-term ibrutinib therapy is likely to reverse the pseudo-exhaustion of T cells and promote the activity of effector T cells in CLL patients by inhibiting ITK activity and reducing the expression of inhibitory receptors (53, 83, 84). Additionally, ibrutinib directly and indirectly blocks the interaction between CLL and T cells by inhibiting cytokine networks and reducing tumor burden (34). Recently, single-cell analysis has shown that ibrutinib significantly increases the expression of cytotoxic genes in CD8^+^ T cells and enhances the function of CTLs with ibrutinib treatment (44, 101).

However, a contradictory finding demonstrated that there was reduced granzyme and IFNγ in CD8^+^ T cells from ibrutinib-treated mice, implying poor cytotoxicity (102). Most studies have shown that ibrutinib treatment promotes the recovery of T-cell cytotoxicity. However, the mechanism of ibrutinib regulating T-cell immunity is not fully clear.

## The impacts of ibrutinib on TCR repertoire diversity

More than 90% of T cells are αβ T cells in the peripheral blood. T-cell receptor (TCR) repertoire diversity is mainly determined by the diversity of the hypervariable complementary determining region 3 (CDR3) of the TCRα and β chains, which specifically recognize antigens presented by major histocompatibility complex (MHC) molecules. A diverse TCR repertoire is used to resist the invasion of various pathogens. However, the TCR repertoire in CLL patients is seriously skewed and exhibits oligoclonal or monoclonal expansion (22, 103, 104) (**Figure 2B**), suggesting a tumor-related antigen-mediated selection (104–106), in parallel with severe impairment of T-cell immunity (105–107). In fact, the pro-tumor and anti-tumor effects of these oligoclonal or monoclonal T cells remain unknown (6, 22, 105). Prior studies have confirmed that there are specific T-cell clones in patients with CLL (6, 104), but they cannot effectively play an anti-tumor role due to their small number and severe immunosuppressive microenvironment (8). Additionally, the diversity of the TCR repertoire is progressively impaired with disease progression and multiple chemotherapy regimens (99, 105). Therefore, the reconstruction of the TCR repertoire may be key to restoring T-cell immune function and further improving the response to antitumor immunotherapy (22, 108).

Yin and colleagues found that the diversity of the TCRβ repertoire could be partially reconstituted in R/R CLL patients after 12 months of ibrutinib treatment, which was closely related to good treatment response and decreased infection rates (22), suggesting that ibrutinib contributes to promoting the reconstruction of the TCR repertoire diversity (**Figure 2B**). However, another study revealed that the clonality of the TCR repertoire increased with ibrutinib treatment in newly diagnosed CLL patients; nevertheless, the clonality disappeared with disease progression (103). These clonally expanded T cells after ibrutinib treatment cannot be excluded as tumor-specific T cells due to the stimulation from CLL-related antigens, which is consistent with previous studies displaying the existence of anti-tumor T-cell clones in CLL patients, suggesting to some extent the recovery of T-cell immunity of patients with CLL (6, 22, 105). The differences in the results of the above two studies may be due to the immune function of the subjects. The former is R/R patients, and the latter is naive patients. Moreover, the difference may also be due to analysis from different standpoints. The former is from the whole TCR repertoire, and the latter is from T-cell immune response in the TCR repertoire.

## Conclusion and future prospects

Tumor microenvironment disorder and T-cell immune dysfunction are the main characteristics of CLL patients (37, 109). Long-term ibrutinib treatment promotes the restoration of immunity, particularly T-cell immunity, consistent with improved clinical outcomes observed in CLL patients (3, 42). Although single-agent ibrutinib has long-term efficacy and tolerability in CLL patients according to an 8-year follow-up (14), combined therapies are still needed to overcome drug resistance, further improve the efficacy of ibrutinib and reduce side effects, such as ibrutinib combined with immunochemotherapy or BCL2 inhibitor venetoclax (110, 111). However, the biggest challenge in the future is to find strategic combinations to overcome T-cell dysfunction, reverse the immunosuppressive environment, and improve the efficacy of targeted immunotherapies in CLL (37, 97, 112). Theoretically, ibrutinib combined with anti-CD20 antibody rituximab (113) or immune checkpoint blockade (ICB) can improve the efficacy of ibrutinib and enhance the anti-tumor effect (103, 114); however, these effects have not been confirmed in the clinic (115–117). Obinutuzumab reportedly appears to have improved antibody-dependent cellular toxicity over rituximab (118).

Recently, increasing studies have demonstrated that ibrutinib has beneficial effects on T-cell-based immunotherapies (23). Both preclinical and clinical studies have confirmed that ibrutinib pretreatment combined with CAR-T cells can promote the implantation and amplification of CAR-T cells and enhance its anti-tumor activity in CLL patients (3, 25, 119), even if in patients with ibrutinib-resistance (3, 25), and decreased toxicity of CAR-T cells (25, 119). There are several possible mechanisms. For instance, long-term ibrutinib treatment regulates the disordered microenvironment (18, 34), decreases the expression of inhibitory molecules in CLL (21, 44, 82, 84), and reverses the limited expansion of T cells (120, 121), particularly naïve-like T cells and stem cell memory-like T cells (122), which play an important role in the expansion and long-term maintenance of CAR-T cells (123, 124). Moreover, ibrutinib can promote the migration of CAR-T cells to the tumor by enhancing CD62L expression (122, 125), which is conducive to the anti-tumor effect of CAR-T cells. Likewise, ibrutinib combined with the bispecific antibody blinatumomab can promote T-cell-mediated anti-tumor effects by inducing T-cell activation and proliferation, triggering cytokine secretion and granzyme release (24, 126, 127). Moreover, emerging targeted therapies, such as CD3/CD20 bispecific antibodies, may provide further combined options (128).

Collectively, based on the effects of ibrutinib on the microenvironment and T-cell immunity, in addition to the benefits of long-term treatment with ibrutinib alone, the combination of ibrutinib with T-cell-based immunotherapies could become a promising treatment with deeper remission and longer survival for CLL patients in the future.

## Author contributions

YL reviewed the literature and wrote the manuscript. YS contributed to manuscript revision. QY designed the review and revised the manuscript. YS and QY equally contributed to this work. All authors contributed to the article and approved the submitted version.

## Funding

This study was supported by the Foundation for Young Teachers’ Basal Research of Zhengzhou University (jc202050015).

## Conflict of interest

The authors declare that the research was conducted in the absence of any commercial or financial relationships that could be construed as a potential conflict of interest.

## Publisher’s note

All claims expressed in this article are solely those of the authors and do not necessarily represent those of their affiliated organizations, or those of the publisher, the editors and the reviewers. Any product that may be evaluated in this article, or claim that may be made by its manufacturer, is not guaranteed or endorsed by the publisher.
